# Hepatitis B virus reactivation and antiviral prophylaxis during lung cancer chemotherapy: A systematic review and meta-analysis

**DOI:** 10.1371/journal.pone.0179680

**Published:** 2017-06-22

**Authors:** Yu-tuan Wu, Xin Li, Zi-li Liu, Zhou Xu, Wei Dai, Ke Zhang, Jiu-song Wu, Bilal Arshad, Kai-nan Wu, Ling-quan Kong

**Affiliations:** Department of Endocrine and Breast Surgery, the First Affiliated Hospital of Chongqing Medical University, Chongqing, China; Academia Sinica, TAIWAN

## Abstract

**Background:**

Antiviral drugs have been recommended as prophylaxis for the reactivation of hepatitis B virus (HBV) infection in cancer patients undergoing chemotherapy. However, screening and antiviral prophylaxis for lung cancer remain controversial because of insufficient evidence.

**Purpose:**

In this study, we investigate the absolute risk for HBV reactivation and the prophylactic effects of antiviral drugs in hepatitis B surface antigen (HBsAg)-positive lung cancer patients during chemotherapy.

**Methods:**

We searched Pubmed, Embase, Cochrane, Web of Science and SinoMed from inception until 28 November 2016, and identified all potential relevant references with or without prophylactic use of antiviral therapy in HBsAg-positive lung cancer patients during chemotherapy. The primary outcome was the incidence of HBV reactivation, the secondary outcomes were the incidence of hepatitis, chemotherapy disruption and mortality.

**Results:**

Eleven studies involving 794 patients were analyzed. The incidences of HBV reactivation in control group and antiviral prophylaxis group ranged from 0% to 38% (median, 21%, 95% CI: 0.17–0.25) and 0% to 7% (median, 4%, 95% CI: 0.02–0.06), respectively. Antiviral prophylaxis had significantly reduced the risk for HBV reactivation (RR, 0.22 [95% CI: 0.13–0.37], p< 0.0001), hepatitis (RR, 0.35 [95% CI: 0.22–0.56], p<0.0001) and chemotherapy disruption (RR: 0.29 [95% CI, 0.15–0.55], p<0.0002) compared to those without antiviral prophylaxis. There was no significant heterogeneity in the comparisons, and a fixed-model was used.

**Conclusion:**

The risks of HBV reactivation and relevant complications are high in HBsAg-positive lung cancer patients receiving chemotherapy, and available evidences support HBV screening for antiviral prophylaxis before initiation of chemotherapy for lung cancer patients.

## Introduction

Hepatitis B virus (HBV) infection is an important public and medical problem. A global systematic review reported that 248 million people were chronically infected with HBV worldwide[1]. This is particularly true in developing areas, especially in Asia, where the infection rates of HBV were of high prevalence[2, 3]. For those with chronic or resolved HBV infection, they are at risk for HBV reactivation (HBVR) when receiving immunosuppressive therapy for various diseases.

As more chemotherapy applications are carried out in cancer patients, HBVR during chemotherapy has become a common problem that cannot be ignored. The increased risk for HBVR had been reported commonly in hematological malignancies[4], additionally, corresponding data in solid tumors were also reported[5]. HBVR had been reported in 20%-50% of patients with chronic HBV infection undergoing cancer chemotherapy or immunosuppressive therapy[6]. The clinical complications of HBVR, varying from asymptomatic hepatitis to life-threatening events[7–9], may cause interruption or early termination of systemic chemotherapy, even HBV-related death, which would cause poor prognosis undoubtedly[10]. Therefore, HBV screening is recommended for cancer patients undergoing chemotherapy, and antiviral prophylaxis is recommended for HBsAg-positive patients[6]. However, despite the risk for reactivation, guidelines in oncology do not recommend universal screening of HBV for patients undergoing chemotherapy for solid tumors because of insufficient evidence[11].

Recent meta-analyses had reported the increased risk for HBVR and effective antiviral prophylaxis during chemotherapy for hematologic tumors and solid tumors (mainly including breast cancer)[4, 12–14], but none had examined HBVR and antiviral prophylaxis during chemotherapy for lung cancer. Lung cancer is the second most frequent cancer diagnosed, and the most common causes of cancer death in both male and female[15, 16]. It has been reported that HBsAg-positive lung cancer patients may experience a high risk for HBVR during cytotoxic chemotherapy[7–10, 17–23].

Therefore, the purpose of this study was designed to investigate the risk for HBVR with or without antiviral prophylaxis and the prophylactic effects of antiviral drugs in reducing the risk for HBVR and the relevant complications in HBsAg-seropositivie lung cancer patients receiving chemotherapy.

## Materials and methods

All the procedures of the systematic review and meta-analysis were conducted according to the MOOSE guidelines(Meta-analysis of Observational Studies in Epidemiology)[24]. We followed an established protocol which had been registered in PROSPERO (International prospective register of systematic reviews)[25], and the record is available on https://www.crd.york.ac.uk/PROSPERO/ (Registration number: CRD42016053110).

### Search strategy

We searched Pubmed, Embase, Cochrane, Web of Science and SinoMed (Chinese Biomedical Database) from inception until 28 November 2016. The literature searches were conducted by a combination of medical subject headings (MeSH) and free terms such as “malignancy”, “neoplasm”, “cancer”, “antiviral”, “hepatitis B virus” and “reactivation”. We had not restricted languages in the course of searching. All the references identified were managed by Endnote. More details are available in S1 Appendix Data.

### Outcomes and definitions

The primary outcomes were the rates of HBVR and the risk ratio (RR) of HBVR comparing the antiviral prophylaxis group with the non-prophylaxis group, and the secondary outcomes were the RR of hepatitis, chemotherapy disruption and mortality. HBVR was considered as an increase in the HBV DNA level of ten-times or more when compared with the baseline level or an absolute increase in the HBV DNA level that exceeded 1×10ˆ9 copies/mL without other systemic infections. Hepatitis was characterized by three times or more increase in serum ALT levels that exceeded the upper limit of normal (ULN) or an absolute rise of ALT to more than 100 U/L. Disruption of chemotherapy was defined as delay 8 or more days in planned chemotherapy regimens and premature termination of chemotherapy. Mortality was defined as death due to HBVR, excluded other causes related mortality, including cancer-related and other systemic infections related mortality. Besides, antiviral prophylaxis was defined as received antiviral medicine daily prior to the commencement of chemotherapy, and continued throughout the course of chemotherapy or last longer.

### Inclusion and exclusion criteria

Eligible studies include randomized and non-randomized clinical studies that had investigated the HBVR with or without antiviral prophylaxis in HBsAg-positive lung cancer patients during chemotherapy. Studies must have reported data on HBVR for lung cancer. Studies were excluded: (1) Case reports, reviews and conference reports. (2) No control group or unable to extract relevant data. (3) Cases coexist of HAV, HCV, HDV or HIV infection.

### Study selection

**Fig 1:**
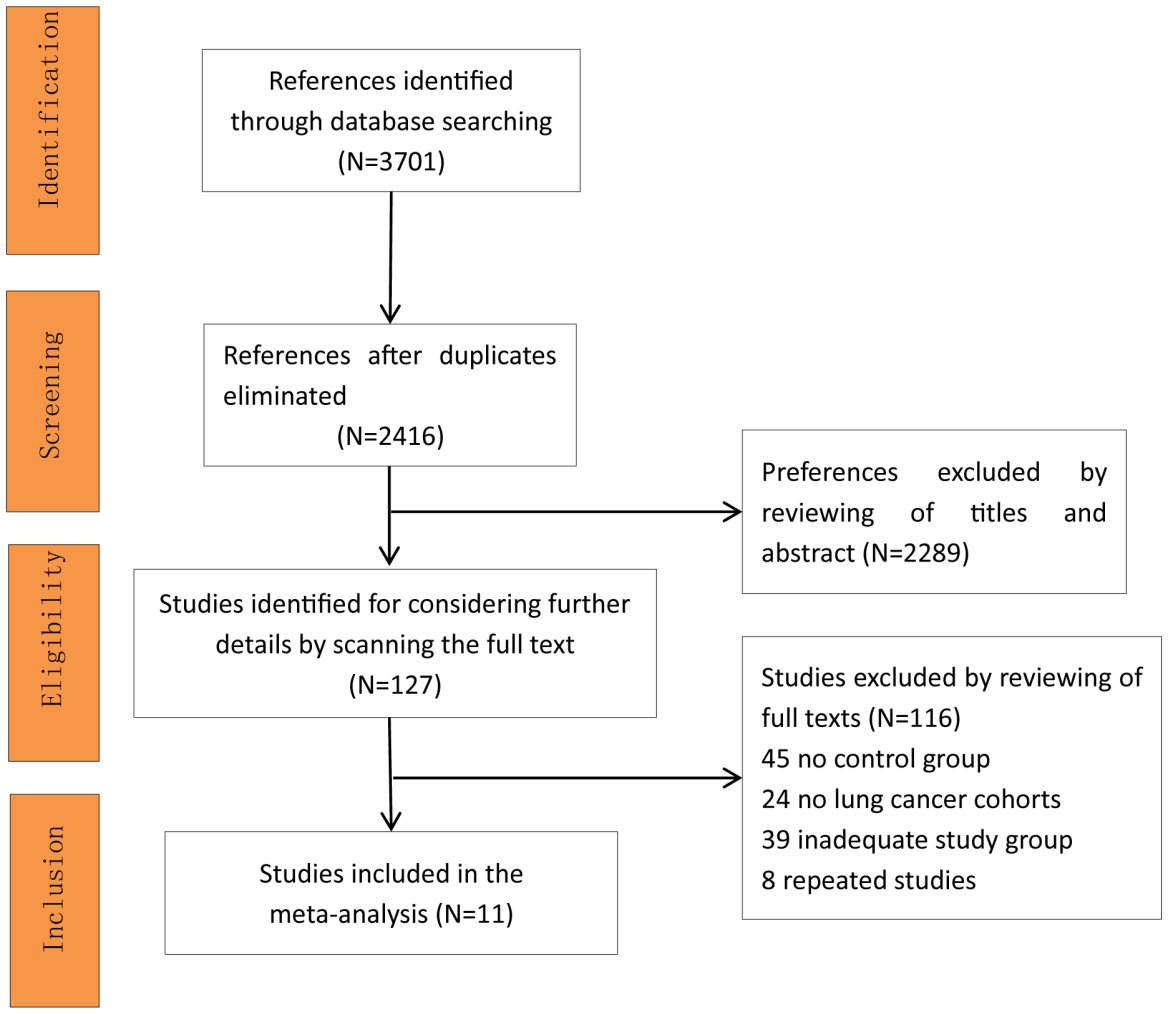
Preferences selection flow diagram.

All 3701 references were identified after searched databases. After duplicates removed, we reviewed of titles and abstracts, and scanned the full text according to the inclusion/exclusion criteria. Finally, total 11 studies[7–10, 17–23] included in the meta-analysis (Fig 1).

### Data collection and quality assessment

Two investigators (X.L and Z.L) independently assessed the eligible studies and extracted data using electronic tables. The following items were summarized: age, gender, type of study design, type of cancer, sample size, basic characteristics HBV DNA level, hepatic function, intervention regimen and outcome.

Two investigators (Z.X and W.D) independently assessed the quality of the included studies using the Newcastle-Ottawa scale (NOS). The NOS has three parameters of quality assessment for prospective or retrospective cohort studies, and assigns a maximum of four points for selection, two points for comparability and three points for outcome. NOS scores of ≥7 points were considered to be high quality studies and 5–6 points were moderate quality. A third investigator (Y.W) was consulted when disagreement arise.

### Statistical analysis

All outcomes were dichotomous variables and presented as absolute risk and RR with 95% confidence intervals (CI). The associated data statistics and potential publication bias evaluation were conducted by the Cochrane Collaboration’s Review Manager Software (RevMan, version 5.3; Oxford, United Kingdom) and the metafor and meta packages in R software (version 3.3.2, R Foundation for Statistical Computing). Probability values were two-sided, and P<0.05 was considered of statistical significance. Statistical heterogeneity was evaluated using the I-squared (I^2^) and chi-squared (χ^2^) tests. I^2^ values of 25%, 50%, 75% indicated low, moderate and high level of heterogeneity, respectively. Data were not pooled if the I^2^ was greater than 40%. A P value of <0.1 for χ^2^ was defined to indicate the presence of heterogeneity. Results were pooled using the maximum likelihood estimation, a fixed effect model was used if no heterogeneity existed[26]. Besides, sensitivity analysis was performed if significant heterogeneity existed. Funnel plots and the Egger’s test of funnel plot asymmetry were used to evaluated publication bias[27].

## Results

### Studies characteristics

In total, 11 studies met the strict inclusion criteria and comprised 794 HBsAg-positive lung cancer patients in the meta-analysis (Fig 1), of which 5 studies were published in Chinese with an English abstract.

We summarized baseline study characteristics (Table 1) and extracted data (S1 Table). Of the 11 studies, 9 were retrospective cohorts and 2 were prospective cohorts. Seven studies were two arms and 4 studies were single arm with non-prophylaxis group. The 11 studies included 6 from mainland China, 2 from Hongkong, 3 from Greece, Turkey and Japan, respectively. All of the 794 patients were HBsAg-seropositive, the median age was 51 years (range 17 to 79 years), with 468 male, 280 female and 46 undescribed. Of the 794 patients, 326 patients received antiviral prophylaxis, of which 211 received lamivudine and 115 received entecavir, besides, 468 patients did not received antiviral prophylaxis.

**Table 1:** Characteristics of the included clinical studies†.

Study, Year (Reference)	Country	Study Design	Type of cancer Testing result of HBsAg†	Total (n)	Group (P/C) †	Sex (M/F) †	Age(y) †	Pre-chemo†			Post-chemo			Intervention		Median follow-up (m) †
								HBV† DNA (Units)	ALT† (IU/L)	TBIL† (Units)	HBV DNA (Units)	ALT (IU/L)	TBIL (Units)	Dosage	Duration	
**Alexopoulos et al, 1999**	Greece	RetrospectiveCohort, Single†	LC† HBsAg+	14	NR/14	NR†	T†: 58(19–78)	NR	NR	NR	NR	NR	NR	NR	NR	12m
**Che et al, 2014**	China	RetrospectiveCohort, Double†	LC HBsAg+	160	80/80	P: 39/41C: 43/37	P: (18–64) C: (17–63)	NR	NR	NR	NR	NR	NR	Entecavir 0.5mg/d†	Start: before chemo. End: 6m after chemo.	NR
**Duan et al, 2009**	China	Prospective Cohort, Double	IIIA NSCLC† HBsAg+	60	30/30	T: 36/24	T: 56(35–71)	NR	P: 34.8±21.5C: 33.5±19.7	NR	NR	P: 75.8±56.4C: 135.2±94.6	NR	LAM† 100mg/d	Start: confirmed diagnosis. End: 6m after chemo.	NR
**Eren et al, 2009**	Turkey	Retrospective Cohort, Double	LC HBsAg+	11	7/4	NR	NR	NR	NR	NR	NR	NR	NR	LAM 100mg/d	Start: before chemo. End: 6m after chemo.	1.5m after chemo
**Lin et al, 2014**	China	RetrospectiveCohort, Double	IIIB~IV NSCLC HBsAg+	258	82/176	P: 49/33C: 107/69	P: 61.5(34–77) C: 59.0(30–79)	>10^4(copies/ml) P: 51pt† C: 116pt ≤10^4(copies/ml) P: 31pt C:60pt	P: 26.5(11–67)C: 31.0(9–88)	P:12.7(4.8–32.4)C:12.3(5.2–30.5)(μmol/L)	NR	NR	NR	LAM 100mg/d	Start: 7d before chemo. End: 3m after chemo.	NR
**Nishida et al, 2013**	Japan	RetrospectiveCohort, Single	LC HBsAg+	2	NR/2	P: NR C: 1/1	P:NR C:58 (52–64)	NR	NR	NR	NR	NR	NR	NR	NR	21m
**Yan et al, 2012**	China	RetrospectiveCohort, Double	LC HBsAg+	76	33/43	P: 27/6 C: 34/9	P: 52 C: 52	NR	NR	NR	NR	NR	NR	LAM	Start: before chemo. End: NR.	NR
**Yeo et al, 2000**	China (Hongkong)	RetrospectiveCohort, Single	LC HBsAg+	8	NR/8	NR	NR	NR	NR	NR	NR	NR	NR	NR	NR	12m
**Yeo et al, 2004**	China (Hongkong)	Prospective Cohort, Single	LC HBsAg+	13	NR/13	NR	NR	NR	NR	NR	NR	NR	NR	NR	NR	2m after chemo
**Zheng et al, 2014**	China	RetrospectiveCohort, Double	LC HBsAg+	122	59/63	P: 40/19C: 53/10	T:47pt>60y 75pt<60y	NR	NR	NR	NR	NR	NR	LAM 100mg/d	Start: within 7d before chemo. End: 6m after chemo.	22m(median 10m)
**Zheng et al, 2016**	China	RetrospectiveCohort, Double	LC HBsAg+	70	35/35	T: 39/31	T: 55.6(26–79)	NR	P: 37.4±7.4C: 37.1±7.8	P:17.8±3.2C:17.6±3.3	NR	P: 71.6±4.9C: 104.6±10.9	P:73.8±7.4C:98.8±11.6	Entecavir 0.5mg/d	Start: 7d before chemo. End: 1m after chemo.	NR

^†^Measurement data were presented as mean± SD or median(range).

Single = single group study; Double = double group study; P = prophylaxis group; C = control group; T = total, prophylaxis group and control group;pt = patient(s); M = male; F = female; d = day(s); m = month(s); y = year(s); HBV = hepatic B virus; ALT = alanine aminotrasferase; TBIL = totol bilirubin level; LC = lung cancer; NSCLC = non-small cell lung cancer; HBsAg = hepatic B virus surface antibody; LAM = lamivudine; chmo = chemotherapy; NR = not reported;

We used NOS to assess the quality of included studies (S2 Table). According to NOS assessments, all of the studies were of moderate or high quality, which indicated that all the included studies were reliable. As for publication bias assessment, symmetrical funnel plot analysis of different outcomes (S1–S3 Figs) and Egger’s tests (P>0.05) showed no significant publication bias was found. No significant between-study heterogeneity was found (I^2^ = 0%), and the fixed-effect model was applied.

### Absolute risk for HBVR

**Fig 2:**
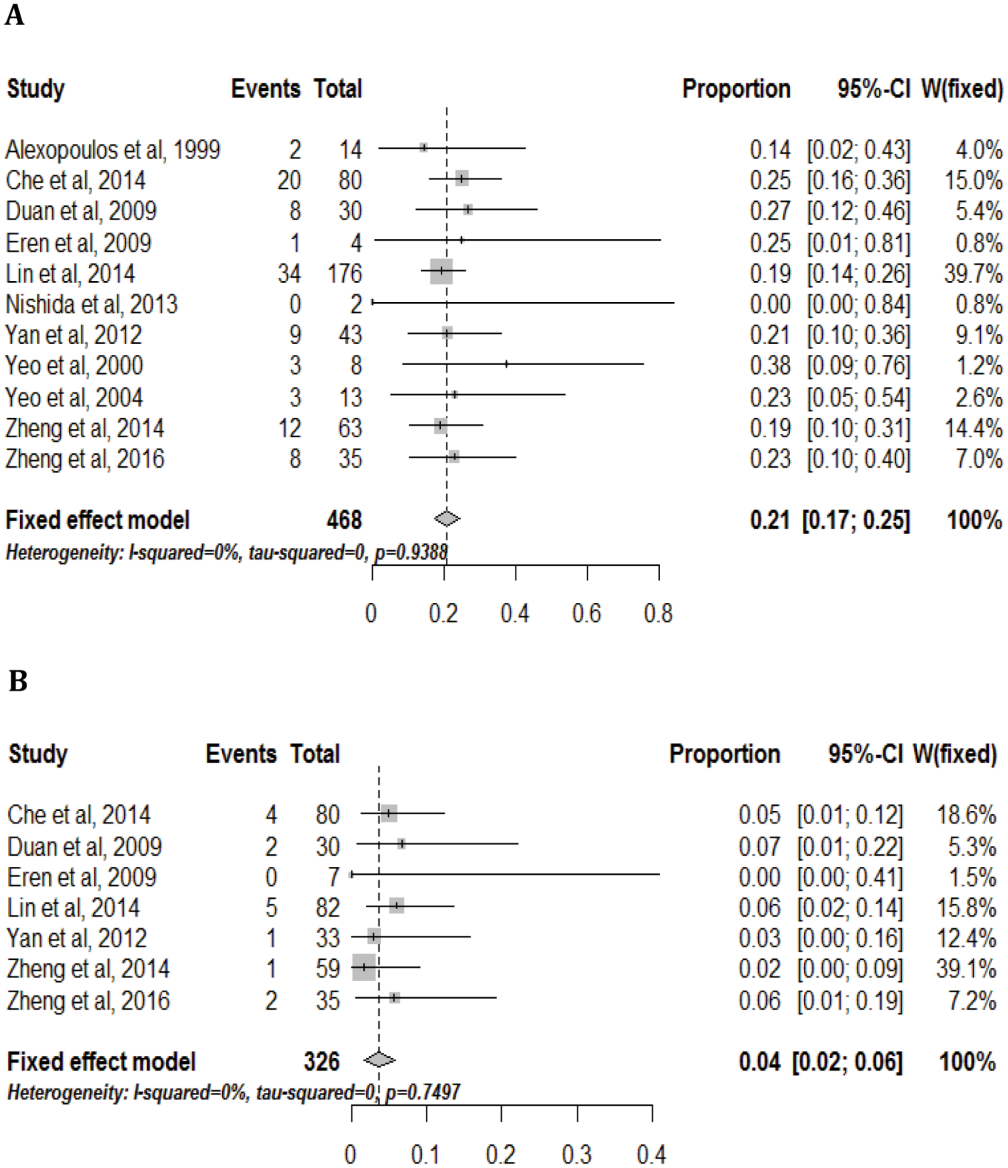
Absolute risk for HBV reactivation in HBsAg-positive lung cancer patients without(2A) or with (2B) antiviral prophylaxis.

In all, 11 studies, included 468 patients, reported HBVR without antiviral prophylaxis. The risk for HBVR ranged from 0% to 38% in 11 studies. The pooled risk for HBVR was 21% (95% CI: 0.17–0.25) (Fig 2A).

All of 7 studies, included 326 patients, reported HBVR with antiviral prophylaxis. The risk was much lower when conducted antiviral prophylaxis, ranged from 0% to 7%, the pooled risk for HBVR was 4% (95% CI: 0.02–0.06) (Fig 2B). The results revealed that patients with antiviral prophylaxis had a much lower risk of HBVR, the pooled risk for HBVR with and without antiviral prophylaxis were 4% and 21%, respectively.

### Absolute risk for HBVR, by area subgroup

When considering the included studies were mostly from mainland China, and China has a high prevalence rate of HBV[3], we examined HBVR without antiviral prophylaxis by area subgroup. In the mainland China group, 6 studies, including 427 patients, reported the risk for HBVR ranged from 19% to 27%, the pooled risk for HBVR was 21% (95% CI: 0.17–0.25) (S4 Fig). In the other regions group, 5 studies, including 2 from Hongkong, 3 from Greece, Turkey and Japan respectively, reported the risk for HBVR ranged from 0% to 38%, the pooled risk for HBVR was 19% (95% CI: 0.07–0.31) (S4 Fig). Beside, we pooled the risk for HBVR in Asians by excluding two studies not from Asia[7, 17], the pooled risk was same with the risk pooled in the mainland China group 21% (95% CI: 0.17–0.25) (S5 Fig).

### Risk ratio of HBVR comparing antiviral prophylaxis with non-prophylaxis

**Fig 3:**
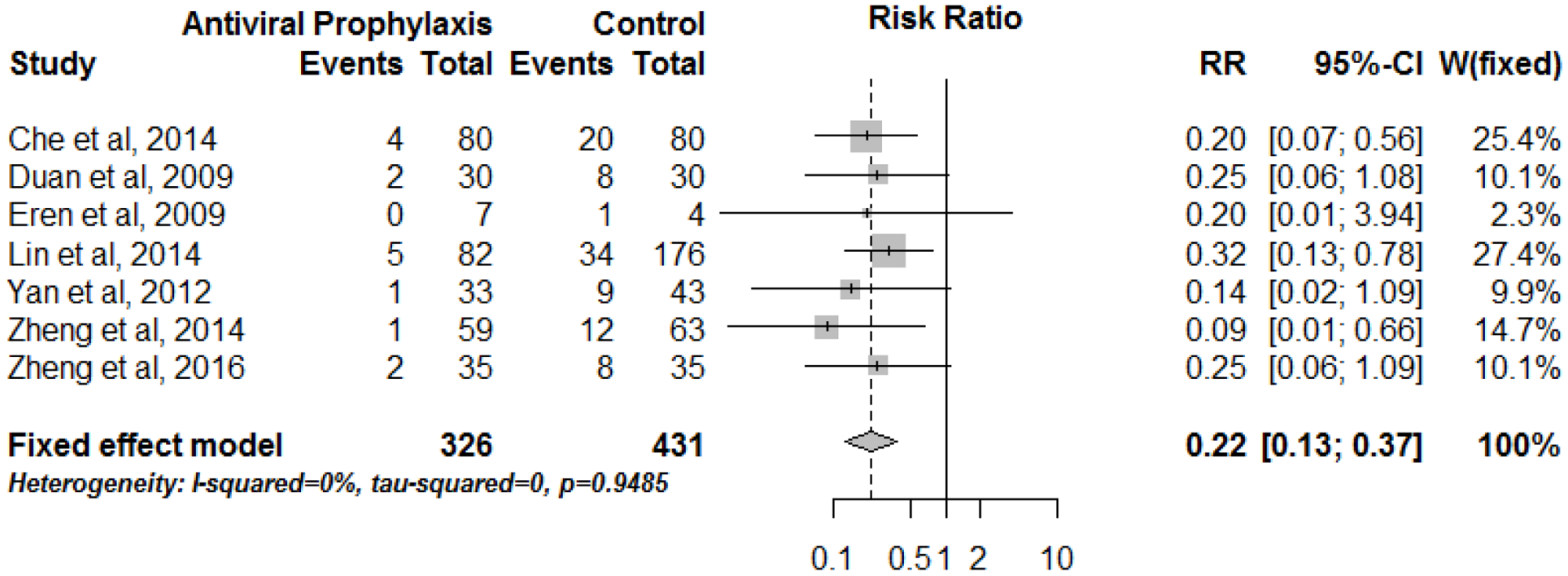
Risk ratio of HBV reactivation comparing antiviral prophylaxis with non-prophylaxis in HBsAg-positive lung cancer patients.

In total, 7 studies had compared HBVR risk in patients receiving antiviral prophylaxis versus non-prophylaxis, including 326 patients in the antiviral prophylaxis group and 431 patients in the non-prophylaxis group. The meta-analysis showed that patients with antiviral prophylaxis had a substantial reduction in the risk of HBVR, the pooled RR was 0.22 (95% CI: 0.13–0.37, p<0.0001) (Fig 3). Further, no single study was found to significantly alter the overall pooled effect.

### Risk ratio of hepatitis comparing antiviral prophylaxis with non-prophylaxis

**Fig 4:**
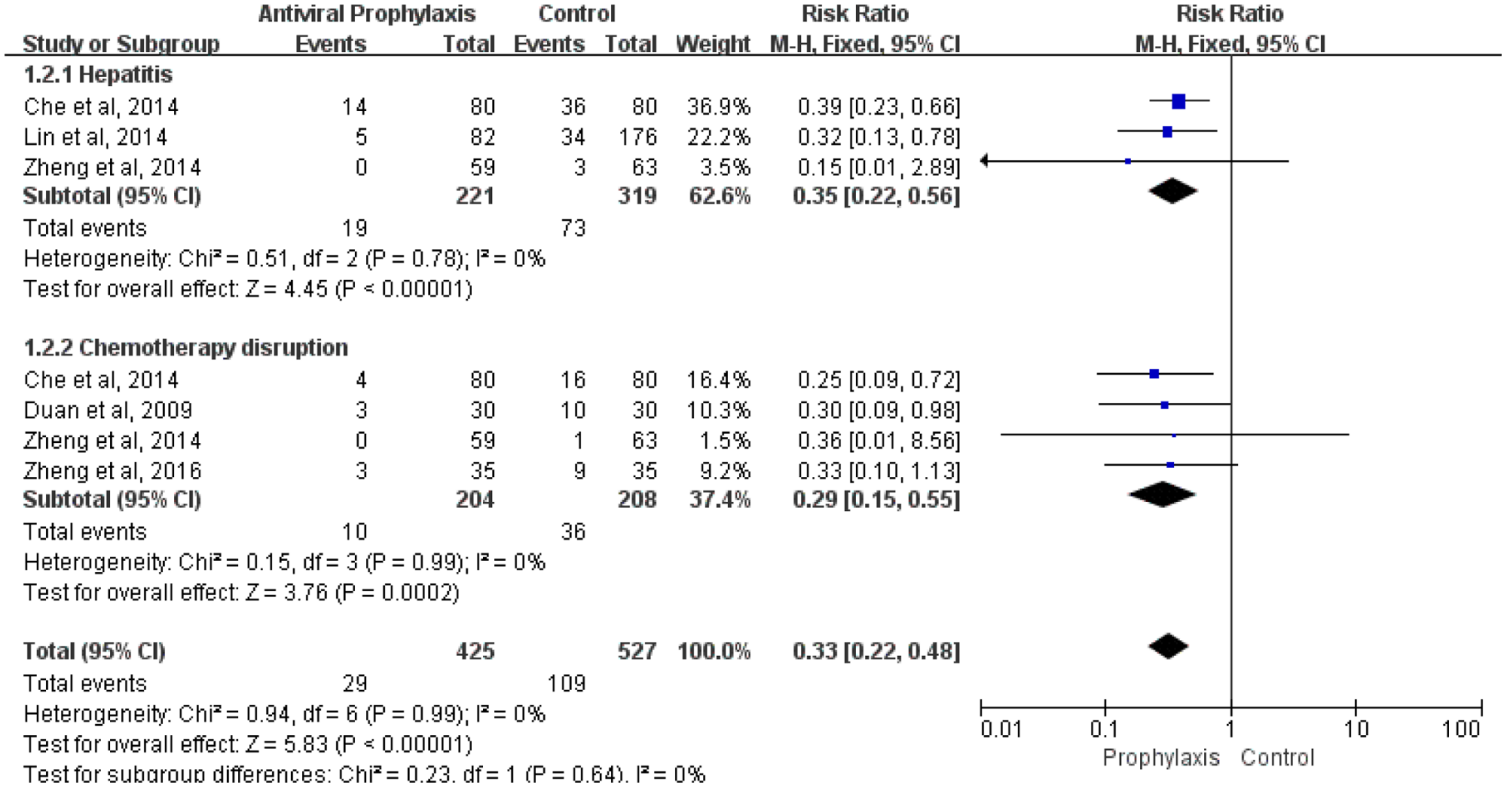
Risk ratio of hepatitis and chemotherapy disruption comparing antiviral prophylaxis with non-prophylaxis in HBsAg-positive lung cancer patients.

Three studies, including 540 patients, reported the incidence of hepatitis. There was a 5% to 45% risk for hepatitis without antiviral prophylaxis, while 0% to 18% risk with antiviral prophylaxis. The incidence of hepatitis in patients with antiviral prophylaxis was significantly lower than without prophylaxis, the pooled RR was 0.35 (95% CI: 0.22–0.56, p<0.0001) (Fig 4).

### Risk ratio of chemotherapy disruption comparing antiviral prophylaxis with non-prophylaxis

Four studies reported on disruptions of chemotherapy. The risk for chemotherapy disruptions ranged from 2% to 33% without antiviral prophylaxis, while the risk ranged from 0% to 9% with antiviral prophylaxis. Meta-analysis revealed that patients with antiviral prophylaxis had a substantial reduction in the risk of chemotherapy disruption, the pooled RR was 0.29 (95% CI: 0.15–0.55, p<0.0002) (Fig 4).

### The mortality

Not enough studies were available to pool for the relevant mortality. Mortality information was reported by three studies[10, 18, 22]. According to Lin et al[10], 4 patients died from HBV-related fulminant hepatitis in non-prophylaxis group, but no significant difference in mortality due to reactivation was noted between patients with or without prophylaxis. Zheng et al[22] reported 92 deaths until the end of 22 months follow-up, but they did not state whether the deaths were HBV-related. Che et al[18] reported no HBV-related death during chemotherapy.

## Discussion

The chemotherapy-induced HBV reactivation may hinder the continuation of the anticancer program. Once HBV reactivation occurred during chemotherapy, it would cause hepatitis or even death which would undoubtedly lead to a negative prognosis of cancer[28]. Several meta-analysis[5, 12–14] noted the risk of HBVR and the effectiveness of antiviral prophylaxis in patients with hematological and solid malignancies, especially in patients with lymphoma and breast cancer. However, we knew little about HBVR and antiviral prophylaxis in lung cancer.

Our meta-analysis shows that the risks of HBVR in the control group and antiviral prophylaxis group were 21% and 4%, respectively. Thus, the risk for HBVR in HBsAg-positive lung cancer patients receiving chemotherapy is very high, and approximately 81% of reactivations can be prevented with antiviral prophylaxis in initiation of chemotherapy. Furthermore, it is also proved that antiviral prophylaxis had substantially lowered the risk for HBVR (RR, 0.22 [95% CI, 0.13–0.37], p< 0.0001), hepatitis (RR, 0.35 [95% CI, 0.22–0.56], p<0.0001) and chemotherapy disruption (RR, 0.29 [95% CI, 0.15–0.55], p<0.0002) compared to those without antiviral prophylaxis. We were not able to pool the mortality due to the incomplete variables from the included studies. Therefore, all available evidences in this study showed that the risk for HBVR and relevant complications are high in HBsAg-positive lung cancer patients receiving chemotherapy, and HBV screening for antiviral prophylaxis should be carried out before initiation of chemotherapy for lung cancer patients.

Appropriate prechemotherapy screening strategy for HBV may be an effective measure to distinguish high-risk populations, especially in areas highly endemic with HBV, such as Asia-Pacific and Africa regions[2, 3, 6]. Although, nucleos(t)ide analogues are effective therapeutic measures for HBVR, the development of HBVR invariably means that anticancer treatment is disrupted, with delay at the least and premature termination in the more severe circumstances[29]. Therefore, antiviral preventing the occurrence of HBVR may provide a more practical approach in managing HBsAg-seropositive patients who require chemotherapy[29]. Previous studies had reported the comparison with and without antiviral prophylaxis in hematological and certain solid malignancies[12, 14, 30]. Our meta-analysis firstly reported the increased risk for HBVR and the benefit of antiviral prophylaxis in HBsAg-seropositive lung cancer patients receiving chemotherapy.

In consideration of most included studies were from Asia, we pooled the incidence of HBVR in the control group after excluding two studies not from Asia[7, 17], and yielded similar results. Due to limited data in the included studies, we were not able to analyze the incidence of HBVR by different chemotherapy regimens. As previously reported, anthracyclines regimens or corticosteroids were more vulnerable to induce HBVR[29–31], both anthracyclines and corticosteroids are commonly used as part of the anticancer treatment and antiemetic premedication for patients who have hematological and solid malignancies[29]. As Paul et al[13] reported, anthracycline-based chemotherapy had a median reactivation risk of 29%, and the platinum-based regimen, which used commonly in lung cancer chemotherapy, had a median reactivation risk of 25%. Additionally, other risk factors for HBVR in patients undergoing chemotherapy have been postulated, including ALT level, baseline HBV DNA level, HBsAg-seropositivity, HBV e antigen seropositivity, precore mutant strain, viral genotype, male sex and young age[28, 32].

Currently, there are no extensive applied screening and prophylaxis recommendation. Several specialty societies[6, 11, 33–36] had recommended HBV screening in HBV-infection high risk patients or if the immunosuppression caused by the treatment is expected to be high. Apart from this general recommendation, the guidelines, given the inaccuracies in ascertaining risks for HBV infection, do not demonstrate unanimous consent in offering HBV screening for all patients undergoing chemotherapy[28]. However, if patients receiving chemotherapy are considered at high risk for HBVR, the screening strategy should be universal[28, 32]. Further, the optimal screening items, initiation and duration of antiviral treatment were controversial. Details of the guidelines are presented in Table 2.

**Table 2:** Hepatitis B virus screening and antiviral prophylaxis strategy prior to immunosuppressive therapy by different major guidelines.

Guidelines	Screening Strategy		Antiviral Prophylaxis Strategy	
	Screening populations	Screening items	Prophylaxis populations	Antiviral duration
**AASLD (Lok etal,2009)**[33]	HBV high risk patients before chemotherapy or immunosuppressive therapy.	HBsAg; antiHBc.	HBV carriers.	**Start**: Before the initiation of cancer chemotherapy or immunosuppressive therapy. **End**: For six months after the completion of chemotherapy or immunosuppressive therapy if baseline HBV DNA<2000UI/mL. Patients with HBV DNA baseline value >2000UI/mL should continue treatment as immunocompetent patients.
**AASLD (Hoofnagle,2009)**[34]	All patients undergoing cancer chemotherapy and marked immunosuppressive treatments.	HBsAg; antiHBc.	HBsAg positive patients should be evaluated for indications for HBV treatment and started on appropriate therapy. Inactive HBsAg carriers should receive antiviral prophylaxis. HBsAg negative and antiHBc positive patients with undetectable HBV DNA should be considered for antiviral treatments if aggressive or long term chemotherapy/ immunosuppression are expected.	**Start**: Before the initiation of cancer chemotherapy or immunosuppressive therapy. **End**: Antiviral therapy should continue for as long as required for management for underlying chronic disease in HBsAg positive patients. In other patients, prophylaxis should continue for at least six months after stopping chemotherapy.
**ASCO (Artz et al,2010)**[11]	Insufficient evidence to determine the benefits and the harms of routine screening in patients undergoing chemotherapy or immunosuppressive treatments. HBV screening requires clinical judgment. May be considered in HBV high risk patients or if highly immunosuppressive therapy is planned.	HBsAg. In some populations, testing for antiHBc should also be considered. Testing for antiHBs in antiHBc positive patients.	In HBV chronic patients, an antiviral treatment should be considered to reduce the risk of HBV reactivation, although evidence is limited.	Screening and/or treating should not delay the initiation of chemotherapy.
**EASL (European Association for the Study of the Liver,2012)**[35]	All candidates for chemotherapy and immunosuppressive treatments.	HBsAg; antiHBc. HBsAg positive candidates for chemotherapy and immunosuppressive therapy should be tested for HBV DNA levels.	HBsAg positive patients should receive preemptive antiviral treatment regardless of HBV DNA level. HBsAg negative and antiHBc positive patients with detectable should be treated as HBsAg positive patients. HBsAg negative and antiHBc positive patients with undetectable HBV DNA should be closely monitored.	**Start**: Preemptive before the start of chemotherapy and immunosuppressive regardless of HBV DNA levels. **End**: 12 months after cessation of chemotherapy and immunosuppressive.
**APASL (Sarin et al,2016)**[6]	Persons needing immunosuppressive or cancer chemotherapy.	Testing should include a serological assay for HBsAg and anti-HBc prior to initiation of treatment. HBsAg-negative patients with positive anti-HBc should be tested for HBV DNA.	HBsAg-positive patients who receive cytotoxic or immunosuppressive therapy. HBsAg-negative, anti-HBc positive patients with detectable HBV DNA should be treated as HBsAg-positive patients. HBsAg-negative, anti-HBc positive patients with undetectable HBV DNA should be followed carefully.	**Start**: Before the start of immunosuppression or chemotherapy. **End**: During therapy and for 12 months after cessation of cancer therapy.
**CDC (Weinbaum et al, 2009)**[36]	All patients needing immunosuppressive treatments should undergo serologic testing.	HBsAg; antiHBc; antiHBs.	All patients HBsAg positive should receive antiviral prophylaxis. Patients antiHBc positive should be closely monitored.	-

**AASLD**: American Association for the Study of Liver Disease; **ASCO**: American Society Clinical Oncology; **EASL**: European Association for the Study of the Liver; **APASL**: Asian Pacific Association for the Study of the Liver; **CDC**: Center for Disease Control. **HBV**: Hepatitis B virus; **HBsAg**: Hepatitis B surface antigen; **antiHBc**: Antibody to hepatitis B core antigen; **antiHBs**: Antibody to hepatitis B surface antigen.

The cost-effectiveness is an important issue when considering universal HBV screening before chemotherapy. According to cost-effectiveness investigation in Australia[37], universal prechemotherapy HBV screening was not cost-effective for patients with solid tumors. However, considering lower than 2% incidence of HBV infection in Australia and higher than 8% prevalence of HBV in Asia, Africa and Pacific Islands[36, 38], the conclusion could not be generalized imprudently. Another cost-effectiveness analysis noted that universal screening and prophylactic to prevent reactivation was the most cost-effective strategy, which would prevent a significant number of reactivations and may extend the lives of cancer patients[39]. However, an appropriate screening strategy remains controversial[32]. Our results support universal screening for lung cancer patients before chemotherapy, although the cost-effectiveness should be evaluated in future studies. However, regarding the moment for testing, it has been suggested that the major benefit is acquired when it is done prior to initiation of chemotherapy[32].

To our knowledge, there has not been meta-analysis systematically investigating the increased risk for HBVR and the prophylactic effects of antiviral drugs in HBsAg-positive lung cancer patients undergoing chemotherapy. We first demonstrated the incidence of HBVR and the prophylactic effect of antiviral treatment in reducing the risk of reactivation and relevant clinical complications in HBsAg-positive lung cancer patients during chemotherapy. The current meta-analysis supports appropriate HBV screening before chemotherapy for lung cancer patients, and antiviral prophylaxis for those with confirmed HBsAg-seropositive.

Our meta-analysis has several limitations. First, included studies were all cohort studies, large prospective randomized trials were lacking. Second, most of study populations were from mainland China, further study should be conducted to investigate other population out of Asia. Third, we were not able to analyze interesting outcomes according to stratification for gender, age, tumor stage and chemotherapy regimens due to unavailable data. Finally, we were not able to assess the optimal initiation and duration of antiviral therapy for those patients due to a lack of relevant data in the included studies. Further investigations should supplement relevant knowledge about these fields.

## Conclusions

In summary, our study demonstrated the increased risk for HBVR and effectiveness of antiviral prophylaxis in HBsAg-positive lung cancer patients undergoing chemotherapy. Our meta-analysis results indicated that the risk for HBVR was substantially higher comparing no antiviral prophylaxis with antiviral prophylaxis, and antiviral prophylaxis significantly reduced the risk for HBVR, hepatitis and chemotherapy disruption. Therefore, we believe that our results support antiviral prophylaxis for parallel patient populations. Besides, we suggest appropriate HBV screening strategy in lung cancer populations before initiation of chemotherapy.

## Supporting information

S1 Fig The funnel plot of eleven studies included in absolute risk analysis in no antiviral prophylaxis group. (TIFF)

S2 Fig The funnel plot of seven studies included in absolute risk analysis in antiviral prophylaxis group. (TIFF)

S3 Fig The funnel plot of seven studies included in risk ratio analysis for HBV reactivation. (TIFF)

S4 Fig Absolute risk for HBV reactivation in HBsAg-positive lung cancer patients without antiviral prophylaxis, by area subgroup. (TIFF)

S5 Fig Absolute risk for HBV reactivation in HBsAg-positive lung cancer patients without antiviral prophylaxis in Asians. (TIFF)

S1 Table The results for primary and secondary outcomes of the clinical studies. (XLSX)

S2 Table Quality assessment of the included sudies using the Newcastle-Ottawa scale in cohort studies. (XLSX)

S1 Appendix Data search strategies. (DOCX)

S1 File PRISMA 2009 checklist. (DOC)
