# Supplementary figures and images for: Hepatitis B virus reactivation and antiviral prophylaxis during lung cancer chemotherapy: A systematic review and meta-analysis
Source: PLoS One. 2017 Jun 22;12(6):e0179680. doi: 10.1371/journal.pone.0179680 (PMC5480953; doi:10.1371/journal.pone.0179680)

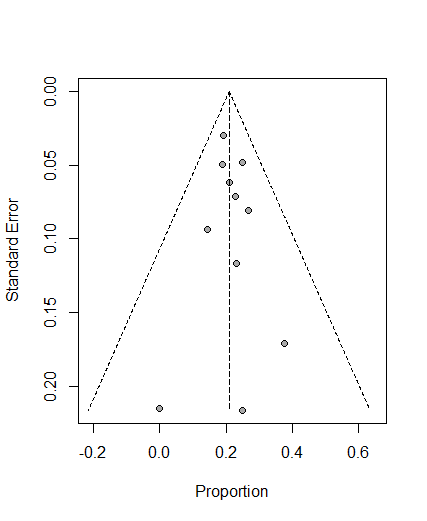

Supplement: S1 Fig — (TIFF) [file pone.0179680.s001.tiff]

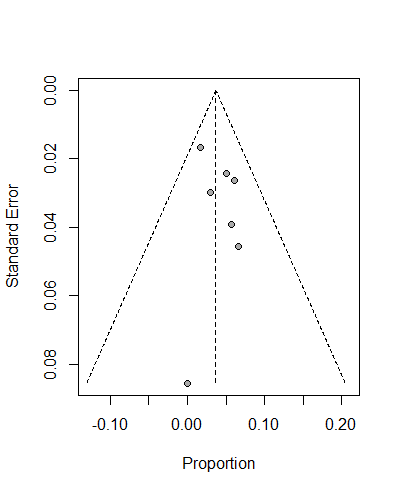

Supplement: S2 Fig — (TIFF) [file pone.0179680.s002.tiff]

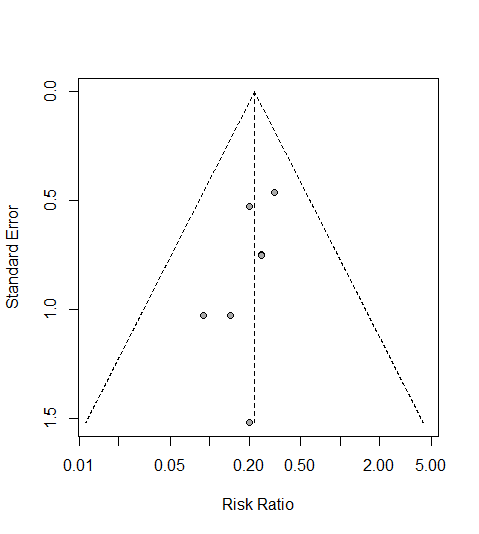

Supplement: S3 Fig — (TIFF) [file pone.0179680.s003.tiff]

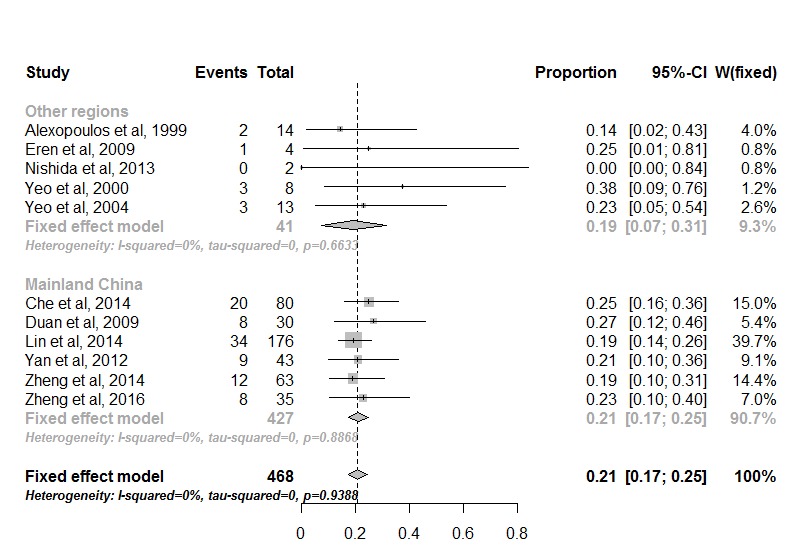

Supplement: S4 Fig — (TIFF) [file pone.0179680.s004.tiff]

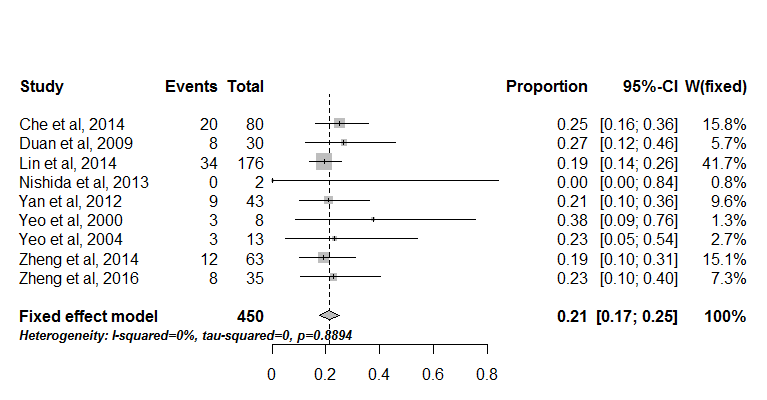

Supplement: S5 Fig — (TIFF) [file pone.0179680.s005.tiff]
