# Supplementary material for: Hepatitis B virus reactivation and antiviral prophylaxis during lung cancer chemotherapy: A systematic review and meta-analysis
Source: PLoS One. 2017 Jun 22;12(6):e0179680. doi: 10.1371/journal.pone.0179680 (PMC5480953; doi:10.1371/journal.pone.0179680)
Supplement: S1 Appendix Data — (DOCX) [file pone.0179680.s008.docx]

**Appendix data – Search Strategies**

1. **Pubmed Search strategy**

**Disease types**

1."neoplasms"[MeSH Terms]

2."Neoplasm"[Title/Abstract]

3."Neoplasms"[Title/Abstract]

4."Malignancy"[Title/Abstract]

5."Malignancies"[Title/Abstract]

6."Malignant"[Title/Abstract]

7."cancer"[Title/Abstract]

8."cancers"[Title/Abstract]

9."tumor"[Title/Abstract]

10."tumors"[Title/Abstract]

11."lung"[Title/Abstract]

12."lungs"[Title/Abstract]

13.or/1-12 (combines all studies)

14."Hepatitis B virus"[MeSH Terms]

15."Hepatitis B"[MeSH Terms]

16."Hepatitis B virus"[Title/Abstract]

17."B virus, Hepatitis"[Title/Abstract]

18."Hepatitis B viruses"[Title/Abstract]

19."virus, Hepatitis B"[Title/Abstract]

20."Hepatitis B"[Title/Abstract]

21."Chronic Hepatitis B"[Title/Abstract]

22.or/14-21 (combines all studies)

**Interventions**

23."Drug Therapy"[MeSH Terms]

24."Therapeutics"[MeSH Terms]

25."Therapeutic"[Title/Abstract]

26."Drug Therapy"[Title/Abstract]

27."Therapy, Drug"[Title/Abstract]

28."Drug Therapies"[Title/Abstract]

29."Therapies, Drug"[Title/Abstract]

30."Chemotherapy"[Title/Abstract]

31."Chemotherapies"[Title/Abstract]

32."Pharmacotherapy"[Title/Abstract]

33."Pharmacotherapies"[Title/Abstract]

34."Treatment"[Title/Abstract]

35."Treatments"[Title/Abstract]

36."Antiviral Agents"[Title/Abstract]

37."Antivirals"[Title/Abstract]

38."Antiviral Drugs"[Title/Abstract]

39.or/1-12 (combines all studies)

**Outcome**

40.reactivation[All Fields]

41. and/13,22,39,40 (combines participants, intervention and outcome studies)

1. **Embase Search strategy**

**Disease types**

1.'hepatitis b virus'/exp

2.'hepatitis b'/exp

3.'hepatitis b virus':ab,ti

4.'b virus, hepatitis':ab,ti

5.'hepatitis b viruses':ab,ti

6.'virus, hepatitis b':ab,ti

7.'hepatitis b':ab,ti

8.'chronic hepatitis b':ab,ti

9. or/1-8 (combines all studies)

10.'neoplasms'/exp

11.'neoplasm':ab,ti

12.'neoplasms':ab,ti

13.'malignancy':ab,ti

14.'malignancies':ab,ti

15.'malignant':ab,ti

16.'cancer':ab,ti

17.'cancers':ab,ti

18.'tumor':ab,ti

19.'tumors':ab,ti

20.'lung':ab,ti

21.'lungs':ab,ti

22. or/10-21 (combines all studies)

**Interventions**

23.'drug therapy'/exp

24.'therapy'/exp

25.'therapeutic':ab,ti

26.'drug therapy':ab,ti

27.'therapy, drug':ab,ti

28.'drug therapies':ab,ti

29.'therapies, drug':ab,ti

30.'chemotherapy':ab,ti

31.'chemotherapies':ab,ti

32.'pharmacotherapy':ab,ti

33.'pharmacotherapies':ab,ti

34.'treatment':ab,ti

35.'treatments':ab,ti

36.'antiviral agents':ab,ti

37.'agents, antiviral':ab,ti

38.'antivirals':ab,ti

39.'antiviral drugs':ab,ti

40.'drugs, antiviral':ab,ti

41. or/23-41 (combines all studies)

**Outcome**

42.reactivation

43. and/9,22,41,42 (combines participants, intervention and outcome studies)

1. **Cochrane Search strategy**

**Disease types**

1.neoplasm:ti,ab,kw

2.malignancy:ti,ab,kw

3.cancer:ti,ab,kw

4.tumor:ti,ab,kw

5.lung:ti,ab,kw (Word variations have been searched)

6. or/1-5 (combines all studies)

7."Hepatitis B virus":ti,ab,kw

8."Hepatitis B":ti,ab,kw

9."Chronic Hepatitis B":ti,ab,kw

10."HBV":ti,ab,kw (Word variations have been searched)

11. or/7-10 (combines all studies)

**Interventions**

12.Therapy:ti,ab,kw

13.Chemotherapy:ti,ab,kw

14.Treatment:ti,ab,kw

15.Antiviral:ti,ab,kw

16.Pharmacotherapy:ti,ab,kw(Word variations have been searched)

17. or/12-16 (combines all studies)

**Outcome**

18.reactivation:ti,ab,kw (Word variations have been searched)

19. and/6,11,17,18 (combines participants, intervention and outcome studies)

1. **Wed of Science**

1. TS=(neoplasm* OR cancer* OR tumor* OR lung*)

Timespan=All years

Search language=Auto

2. TS=(Hepatitis B OR Hepatitis B virus OR HBV)

Timespan=All years

Search language=Auto

3. TS=(Therapy OR Chemotherapy OR Antiviral OR Pharmacotherapy)

Timespan=All years

Search language=Auto

4. TS=reactivation

Timespan=All years

Search language=Auto

5. and/1,2,3,4 (combines participants, intervention and outcome studies)

**5. SinoMed (Chinese)**

|  | 1."肿瘤"[常用字段:智能]  2."乙型肝炎"[常用字段:智能]  3."治疗"[常用字段:智能]  4."激活"[常用字段:智能] |
| --- | --- |

5. and/1,2,3,4 (combines participants, intervention and outcome studies)
